# GRHL2 suppression of NT5E/CD73 in breast cancer cells modulates CD73-mediated adenosine production and T cell recruitment

**DOI:** 10.1016/j.isci.2024.109738

**Published:** 2024-04-12

**Authors:** Bircan Coban, Zi Wang, Chen-yi Liao, Klara Beslmüller, Mieke A.M. Timmermans, John W.M. Martens, Jasmijn H.M. Hundscheid, Bram Slutter, Annelien J.M. Zweemer, Elsa Neubert, Erik H.J. Danen

**Affiliations:** 1 Leiden Academic Center for Drug Research, Leiden University, Leiden, the Netherlands; 2 Department of clinical laboratory, Tianjin Medical University Cancer Institute and Hospital, National Clinical Research Center for Cancer, Tianjin, China; 3 Department of Medical Oncology, Erasmus MC Cancer Institute, Erasmus University Medical Center, Rotterdam, the Netherlands

**Keywords:** Molecular biology, Immunology, Cell biology, Cancer

## Abstract

Tumor tissues often contain high extracellular adenosine, promoting an immunosuppressed environment linked to mesenchymal transition and immune evasion. Here, we show that loss of the epithelial transcription factor, GRHL2, triggers NT5E/CD73 ecto-enzyme expression, augmenting the conversion of AMP to adenosine. GRHL2 binds an intronic *NT5E* sequence and is negatively correlated with NT5E/CD73 in breast cancer cell lines and patients. Remarkably, the increased adenosine levels triggered by GRHL2 depletion in MCF-7 breast cancer cells do not suppress but mildly increase CD8 T cell recruitment, a response mimicked by a stable adenosine analog but prevented by CD73 inhibition. Indeed, NT5E expression shows a positive rather than negative association with CD8 T cell infiltration in breast cancer patients. These findings reveal a GRHL2-regulated immune modulation mechanism in breast cancers and show that extracellular adenosine, besides its established role as a suppressor of T cell-mediated cytotoxicity, is associated with enhanced T cell recruitment.

## Highlights

• GRHL2 suppresses NT5E/CD73 expression in breast cancer cells
• Loss of GRGL2 triggers CD73-mediated adenosine production
• Increased adenosine production does not inhibit T cell migration
• CD73 expression correlates with increased CD8 T cell presence in breast cancer

## Graphical abstract

**Figure:**
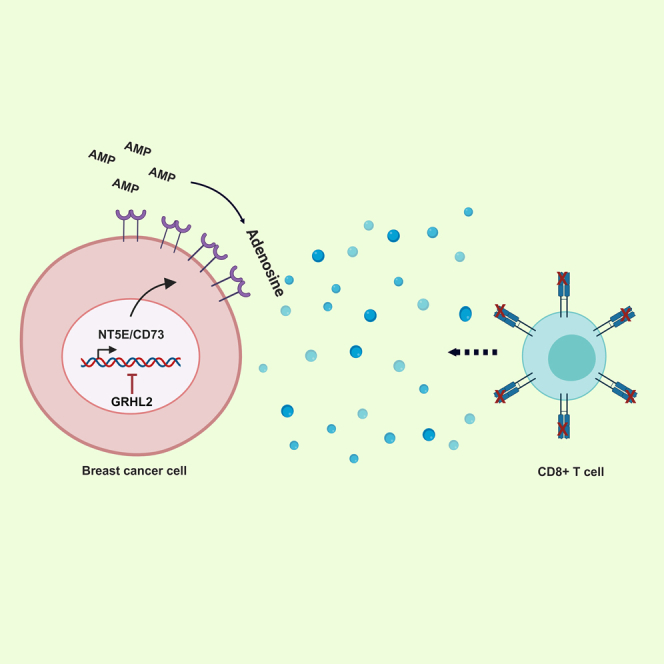

## Introduction

In solid tumors, the interaction between cancer cells and the surrounding tumor microenvironment (TME) regulates cancer growth and metastasis.^1^^,^^2^^,^^3^ The TME is complex and includes altered functionality of extracellular matrix, fibroblasts, and vascular cells. In this tumor-reactive stroma environment, various types of immune cells are affected. On the one hand, this involves crosstalk of tumor cells with myeloid cells such as macrophages and neutrophils, and cancer-associated fibroblasts, creating an inflammatory TME that drives tumor progression.^4^^,^^5^^,^^6^ On the other hand, tumors escape recognition and killing by the immune system by suppressing the activity of T cells and natural killer (NK) cells through a plethora of mechanisms, including the development of an immunosuppressed TME.^4^^,^^7^^,^^8^

One mechanism underlying the emergence of an immunosuppressed niche involves the accumulation of adenosine in the TME.^9^ During physiological healing of wounded or infected tissues, adenosine triphosphate (ATP) released by damaged cells triggers inflammation by binding to excitatory ATP receptors activating T cells and other immune cell types. This response is kept in check by a negative feedback loop, in which ATP and adenosine diphosphate (ADP) are converted to adenosine monophosphate (AMP), which is further converted to adenosine, which binds inhibitory G-protein coupled receptors (GPCRs) on immune cells to dampen the inflammatory response.^10^ In tumors, prolonged elevated levels of extracellular adenosine in the TME suppress immune cell activation and effector functions, thereby causing immune escape and therapy resistance.^9^^,^^11^ In addition to indirect mechanisms such as leakage from necrotic cells in hypoxic areas, the accumulation of adenosine in the TME may be a consequence of genetic changes that alter nucleotide metabolism in tumor cells.

Nucleotide metabolism leading to the production of extracellular adenosine involves the conversion of ATP and ADP to AMP by ecto-enzymes, including ectonucleoside triphosphate diphosphohydrolase-1 (ENTPD1/CD39)^12^ followed by the subsequent conversion of AMP to adenosine, mainly by the ecto-5′-nucleotidase, NT5E/CD73.^13^ CD39 is a transmembrane protein whereas CD73 is linked to glycosylphosphatidyl inositol (GPI) in the plasma membrane and can be shed from the membrane through proteolytic cleavage or GPI hydrolysis.^13^ Given their key role in the creation of an immunosuppressed TME, CD39 and CD73 represent candidate targets for cancer immunotherapy, and their potential has been established in preclinical models.^14^^,^^15^^,^^16^^,^^17^^,^^18^^,^^19^^,^^20^^,^^21^^,^^22^ Increased levels of CD73 expression have been reported in multiple solid tumor types,^23^^,^^24^^,^^25^^,^^26^^,^^27^ but it is incompletely understood how tumor cells may modulate expression of this ecto-enzyme. Tumor cells display plasticity, and carcinomas typically contain populations of tumor cells with different epithelial versus mesenchymal characteristics.^2^ The transition to a more mesenchymal state has been associated with immune evasion in breast and other carcinomas.^28^^,^^29^ Recent studies have shown that epithelial-mesenchymal transition (EMT) can modulate CD73, which may involve activation of transforming growth factor β (TGF-β) signaling and the SNAIL transcription factor and contributes to immune suppression.^30^^,^^31^^,^^32^

Grainyhead-like 2 (GRHL2) is a critical transcription factor for development and function of epithelial tissues that competes with mesenchymal transcription factors such as ZEB and SNAIL to suppress EMT.^33^^,^^34^^,^^35^^,^^36^^,^^37^^,^^38^^,^^39^ We have previously identified GHRL2-regulated genes in breast cancer cells using conditional knockout (KO) cells, chromatin immunoprecipitation sequencing (ChIP-seq), and Bru-seq.^40^^,^^41^ Here, we identify the *NT5E* gene as a GRHL2 target and reveal how GRHL2 depletion induces NT5E/CD73 expression and, consequently, triggers extracellular adenosine production. We find that GRHL2 and CD73 are inversely correlated in a panel of human breast cancer cell lines and human breast cancer patients. We show that, in addition to its previously established role as a suppressor of T cell-mediated cytotoxicity, CD73-mediated adenosine production in fact leads to an increase in T cell recruitment and, in agreement, NT5E expression is positively rather than negatively associated with T cell infiltration in breast cancer patients.

## Results

### Loss of GRHL2 upregulates NT5E/CD73 expression in MCF-7 cells

**Figure 1:**
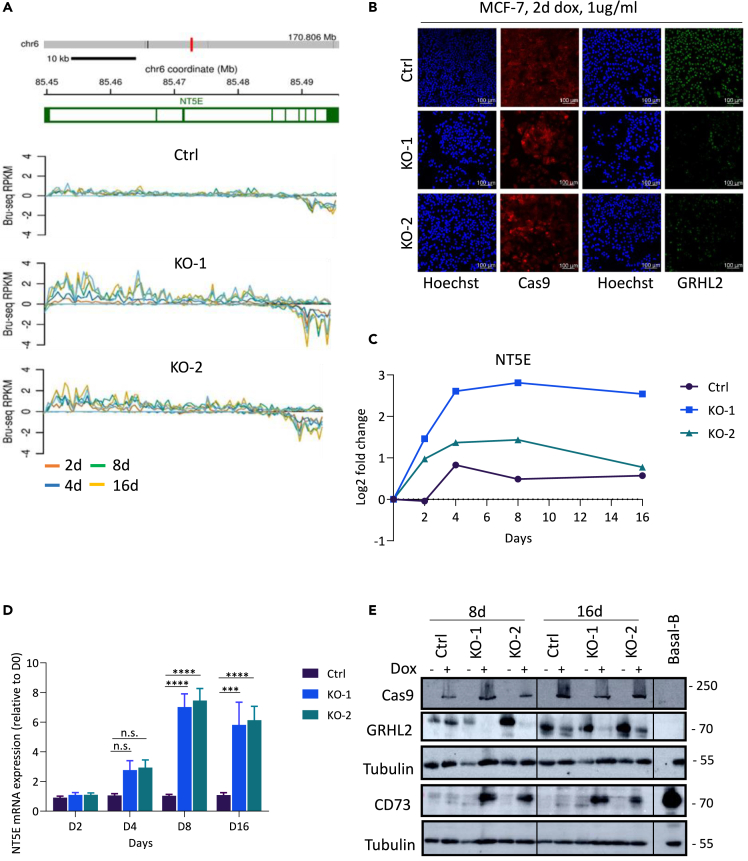
Loss of GRHL2 upregulates CD73 expression in MCF-7 cells (A) Bru-seq reads of nascent NT5E mRNA in an MCF-7 conditional GRHL2 KO model. Graphs are shown for a control sgRNA (Ctrl) and 2 GRHL2 sgRNA models (KO-1 and 2), and colors represent the indicated time points after doxycycline-induced GRHL2 deletion. The reference sequence annotation is shown above with exons in green blocks. (B) Immunofluorescence images of HOECHST (blue), Cas9 Ab (red), or GRHL2 Ab (green) for Ctrl or GRHL2 KO MCF-7 cells after 48 h 1 μg/ml doxycycline treatment. (C) Graph showing log2 fold changes of nascent NT5E mRNA for the indicated time points after doxycycline exposure in Ctrl or GRHL2 KO MCF-7 cells. (D) RT-qPCR analysis showing changes in total NT5E mRNA expression at the indicated time points after doxycycline-induced GRHL2 deletion. Values were normalized to the untreated samples for each time point. Data analyzed using 2–ΔΔCt method. Mean and SD of three biological replicates is shown. (two-way ANOVA test; n.s., non-significant; ∗∗∗*p* < 0.001; ∗∗∗∗*p* < 0.0001). (E) Western blot analysis of Cas9, GRHL2, and CD73 in MCF-7 control sgRNA (Ctrl) and 2 GRHL2 sgRNA models (KO-1 and KO-2) at the indicated time points after doxycycline induced GRHL2 deletion. Tubulin serves as a loading control. One out of three biological replicates shown. MDA-MB-231 basal B cells serve as positive control for CD73 expression.

We explored GRHL2-regulated genes identified by nascent RNA Bru-seq in an MCF-7 conditional GRHL2 KO model. We previously developed this model using doxycycline-induced Cas9 expression to trigger GRHL2 depletion.^41^ One of the genes whose transcription was upregulated in response to loss of GRHL2 was *NT5E*. Sequence reads of NT5E nascent mRNA were mapped to the NT5E genomic sequence in control (Ctrl) sgRNA, GRHL2 sgRNA#1 (KO-1), and GRHL2 sgRNA#2(KO-2) samples (Figures 1A and 1B). An increase in the rate of synthesis of NT5E mRNA was observed upon GRHL2 deletion for both KOs, and this rate decreased at the 3′end of the transcript, indicative of nascent mRNA degradation. Normalized log2 fold changes showed that nascent NT5E mRNA was induced within the first 48 h of doxycycline-induced GRHL2 depletion and increased transcription was maintained for at least 16 days for one sgRNA (KO-1) whereas a second sgRNA (KO-2) showed a gradual return to baseline in this experiment (Figure 1C).

We confirmed the Bru-seq data by quantitative reverse-transcription PCR (RT-qPCR) on RNA samples extracted from the GRHL2 KO MCF-7 cells (Figure 1D). Total NT5E mRNA levels were increased in response to GRHL2 deletion starting from day 4 and remaining high at 8 and 16 days after loss of GRHL2. Increased NT5E mRNA levels were accompanied by an induction of the NT5E/CD73 protein (Figure 1E). CD73 protein expression emerged in GRHL2-depleted cells at 8 and 16 days after doxycycline treatment in both GRHL2 KO-1 and KO-2 models but not in the Ctrl model. A basal B cell line expressing CD73 served as a positive Ctrl. Together, these findings demonstrated that GRHL2 controls expression of the NT5E gene and, consequently, the CD73 protein.

### NT5E/CD73 levels are inversely correlated with GRHL2 in breast cancer cell lines and breast cancer patient tumor samples

**Figure 2:**
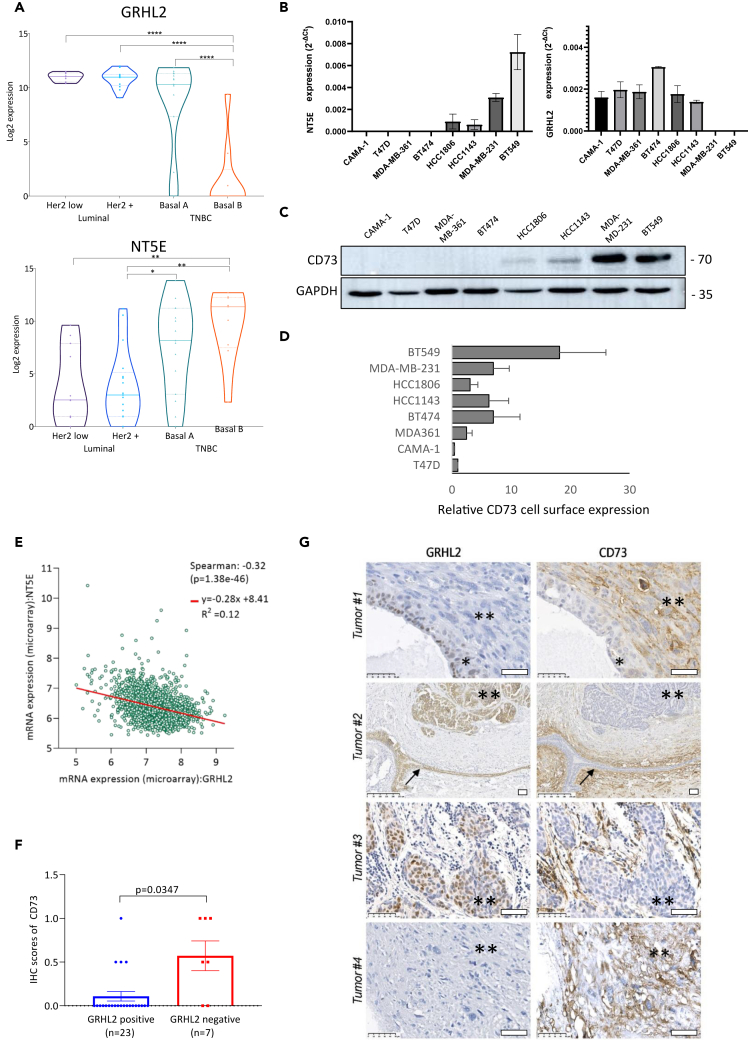
NT5E/CD73 levels are inversely correlated with GRHL2 in breast cancer subtypes and breast cancer patient tumor samples (A) Violin plots showing gene expression levels of GRHL2 and NT5E based on RNA-seq data for 52 human breast cancer cell lines grouped according to the indicated subtypes. ∗*p* < 0.05; ∗∗*p* < 0.01; ∗∗∗∗*p* < 0.0001. *p* values calculated using one-way ANOVA as described in a previous study.^42^ (B) RT-qPCR analysis showing CD73 (left panel) and GRHL2 mRNA expression for the indicated cell lines. Data analyzed using 2–ΔCt method for the gene of interest corrected for GAPDH control. Mean and SD of two experiments performed in triplicate is shown. (two-way ANOVA test). (C) Western blot analysis of CD73 and GAPDH loading control in the indicated cell lines. One experiment of 2 is shown. (D) Flow cytometry analysis of CD73 surface expression in the indicated cell lines. Mean and SD of two experiments performed in duplicate is shown. (E) Correlation between gene expression levels of NT5E and GRHL2 in breast cancer tumors using METABRIC dataset. Correlation scores and *p* values shown as determined in cBioPortal. (F) CD73 score derived from IHC, on whole slides of a series of metaplastic (TNBC) and high and low GRHL2 mRNA tumors, separated for GRHL2 IHC positive and GRHL2 IHC negative cases. Mean and SEM is shown. *p* value calculated using non-parametric t test with unequal variance. (G) Representative IHC images for GRHL2 and CD73 in breast cancer tissues. Tumor 1, 2, and 4 are metaplastic tumors. **Tumor 1** containing ∗area with non-invasive tumor cells staining positive for GRHL2 and negative for CD73. ∗∗area with stroma containing invasive tumor cells staining negative for GRHL2 and positive for CD73. **Tumor 2** Arrow indicates milk duct with GRHL2 positive/CD73 negative epithelial cells surrounded by GRHL2 negative/CD73 positive stroma. ∗∗area with invasive tumor cells staining positive for GRHL2 and negative for CD73. **Tumor 3** mRNA high tumor containing ∗∗area with stroma containing invasive tumor cells staining positive for GRHL2 and negative for CD73. **Tumor 4** ∗∗area containing invasive tumor cells staining negative for GRHL2 and positive for CD73. Bars, 50 μm; note that magnification is higher for tumor 1, 3, and 4 versus tumor 2.

We next addressed whether the inverse correlation between GRHL2 and NT5E identified in the MCF-7 conditional KO models was observed in a larger series of breast cancer cell lines. For this purpose, RNA sequencing (RNA-seq) data from 52 human breast cancer cell lines representing distinct breast cancer subtypes were explored.^42^ Indeed, GRHL2 mRNA was highly expressed in Her2 low and Her2+ luminal lines and, with more variation, in TNBC basal A breast cancer cell lines while it was not or lowly expressed in TNBC basal B cell lines (*p* < 0.0001); NT5E showed an opposite pattern (Figure 2A). NT5E mRNA expression was somewhat increased in TNBC basal A cell lines as compared to the luminal breast cancer cell lines (*p* < 0.05) and showed a sharp further increase in TNBC basal B cell lines (*p* < 0.001). For basal A, NT5E expression showed a wide distribution, indicating that GRHL2/NT5E double-positive lines as well as GRHL2-positive/NT5E-negative lines may be present in this subtype. Immunohistochemistry (IHC) confirmed the inverse correlation between GRHL2 and CD73 in selected luminal versus basal B cells (Figure S1). The inverse correlation between GRHL2 and NT5E/CD73 expression was further established in a series of breast cancer cell lines representing Her2 negative luminal (CAMA-1 and T47D), Her2+ luminal (MDA-MB-361 and BT474), TNBC basal A (HCC1806 and HCC1143), and TNBC basal B cell lines (MDA-MB-231 and BT549). GRHL2 mRNA decreased while NT5E mRNA emerged in the basal B cell lines (Figure 2B). In agreement, CD73 protein expression in this series was not detected in luminal cell lines, weakly expressed in basal A cell lines, and strongly expressed in the GRHL2-negative basal B cell lines (Figure 2C). Notably, CD73 cell-surface expression as detected by flow cytometry was more heterogeneous with an increase in BT549 but not MDA-MB-231 basal B cells as compared to luminal and basal A cells (Figure 2D). This difference between total cellular versus cell surface detected CD73 protein may be related to differences in CD73 shedding from the membrane.^13^

To determine the clinical relevance of this inverse correlation, we used the publicly available dataset METABRIC, containing targeted sequencing mRNA data of 1,904 primary breast cancer samples, and performed a co-expression analysis using cBioPortal. The co-regulation analysis revealed a significant negative relation between GRHL2 and NT5E mRNAs in breast cancer patient tumors with a Spearman value of −0.32 (*p* < 0.001) (Figure 2E). We further investigated the relation between GRHL2 and CD73 protein expression levels by IHC on samples of human breast cancer tissue, choosing a set of 10 GRHL2-high, 10 GRHL2-low (based on RNA-seq), and 10 metaplastic breast adenocarcinoma patient tumors as these were expected to be heterogeneous. Scoring GRHL2 nuclear staining versus CD73 membrane staining on these TMAs further confirmed a negative correlation (Figures 2F and 2G). Tumors often showed areas with GRHL2-negative and areas with positive cells. GRHL2-positive tumors or tumor areas were mostly CD73 negative; GRHL2-negative tumors or tumor areas showed a mixed pattern for CD73. The presence of CD73 in vessels, fibroblasts, and inflammatory cells in some cases prevented accurate assessment of CD73 expression in tumor cells. Taken together, in agreement with the findings in the MCF-7 conditional KO model, these findings indicated that NT5E/CD73 is negatively correlated with GRHL2 in human breast cancer cell lines and breast cancer patients.

### GRHL2 binds NT5E gene in multiple luminal and basal A breast cancer cell lines

**Figure 3:**
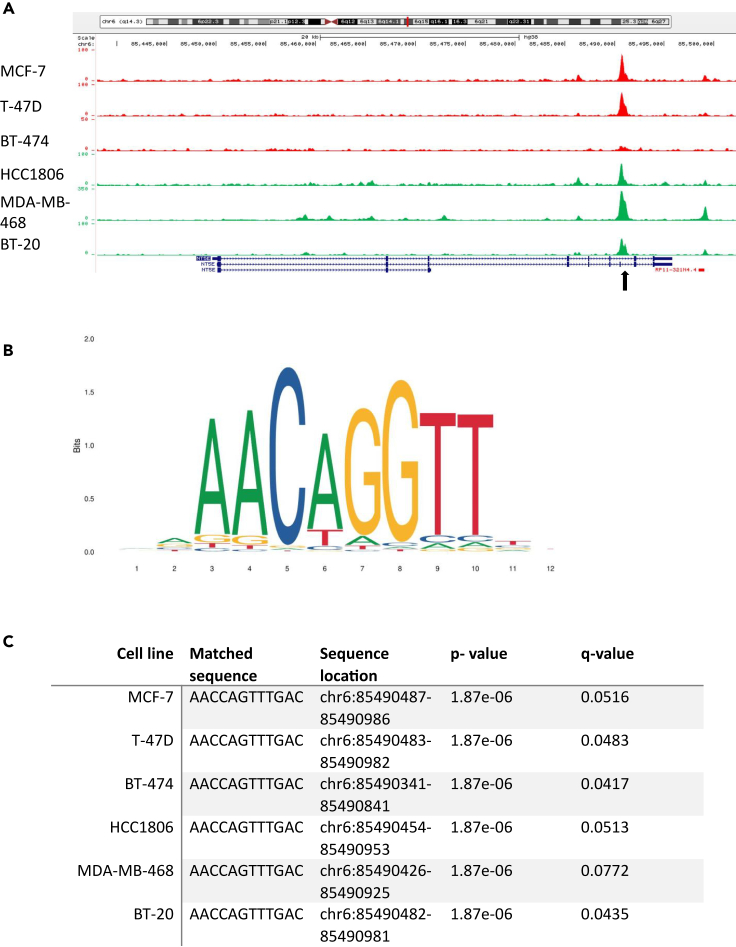
Conserved GRHL2 binding site in the NT5E gene across luminal and basal A breast cancer cell lines (A) GRHL2 ChIP tracks showing its interactions along the NT5E DNA in the indicated luminal (labeled red) and basal A cell lines (labeled green). Note that track heights use different scales. Annotation of the reference sequence is displayed below with exons in blue bars. (B) GRHL2 binding motif identified in GRHL2 ChIP-seq peaks on NT5E DNA using MEME ChIP on data retrieved from the JASPAR database. Y axis shows frequency matrix of each base occurrence. (C) Table showing locations and frequency of the GRHL2 motif in the NT5E gene for the indicated breast cancer cell lines as determined using FIMO. Motif occurrence measured with log-odds scores and converted into *p* values; q values calculated using Benjamini and Hochberg method.

Having established an inverse correlation between GRHL2 and NT5E/CD73, we asked whether the *NT5E* gene could be subject to direct transcriptional regulation by GRHL2 in breast cancer. Therefore, we analyzed our recent ChIP-seq data exploring genome-wide binding sites of GRHL2 in three luminal human breast cancer cell lines (MCF-7, T47D, and BT474) and three basal A human breast cancer cell lines (HCC1806, BT20, and MDA-MB-468).^40^^,^^41^ Analysis of ChIP-seq tracks along the *NT5E* gene located on chromosome 6 revealed a GRHL2 binding site in intron 6 (intron 7 in a *NT5E* gene variant that has a short exon inserted upstream of the GRHL2 peak) that was conserved among all six cell lines (Figure 3A). Further analysis of these peaks using MEME ChIP identified a core GRHL2 binding motif (AACC[A/C/G]GTT) (Figure 3B). The occurrence of the GRHL2 motif in the region occupied by GRHL2 in intron 6 was also confirmed in all six breast cancer cell lines using FIMO^43^ (Figure 3C). Lastly, similar to the large majority of GRHL2 binding sites^41^ no AGGTCAnnnTGACCT ER⍺ motif was detected in a −1,000 to +1,000 nucleotide stretch around the GRHL2 peak. These data demonstrated that GRHL2 interacts with the *NT5E* gene and the inverse relation between GRHL2 and NT5E/CD73 in breast cancer may involve GRHL2-mediated negative transcriptional regulation.

### GRHL2 regulates CD73-mediated extracellular adenosine production

**Figure 4:**
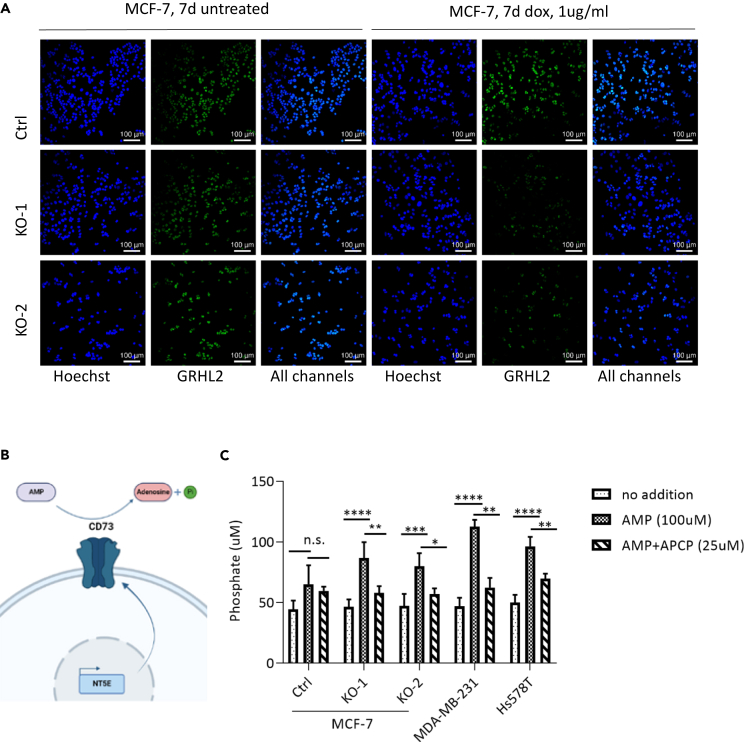
GRHL2 regulates CD73-mediated extracellular adenosine production (A) Immunofluorescence staining showing GRHL2 loss at 7 days after doxycycline-mediated Cas9 induction in sgGRHL2 cells (KO-1 and KO-2) but not sgCtrl cells for the experiment shown in C. Green, GRHL2 Ab; Blue, Hoechst. (B) Cartoon displaying the enzymatic conversion of AMP into adenosine by CD73 resulting in the production inorganic phosphate. Image created using BioRender. (C) Inorganic phosphate concentration measured by malachite green assay in culture supernatants from the MCF-7 conditional KO model taken in parallel to images shown in A and basal B positive control cell lines MDA-MB-231 and Hs578T. Cells were incubated in absence or presence of 100 μM AMP (substrate) with or without 25 μM CD73 inhibitor, APCP for 125 min. Mean ± SD of four biological replicates is shown (two-way ANOVA test; n.s., non-significant; ∗*p* < 0.05; ∗∗*p* < 0.01; ∗∗∗*p* < 0.001; ∗∗∗∗*p* < 0.0001).

CD73 is a cell-surface ecto-nucleotidase that catalyzes the conversion of extracellular AMP into adenosine.^13^ As we observed negative regulation of NT5E/CD73 by GRHL2, we next investigated whether loss of GRHL2 led to an increase in CD73-mediated adenosine production in MCF-7 cells. After 7 days of doxycycline exposure, we observed a strong reduction of GRHL2 protein expression in the GRHL2 KO-1 and GRHL2 KO-2 models whereas no alterations in GRHL2 protein expression were detected in the Ctrl sgRNA model condition (Figure 4A). Parallel samples were generated for the analysis of adenosine production using malachite green, which detects the concentration of inorganic phosphate in the supernatant that accumulates as a result of the conversion of AMP into adenosine (Figure 4B). Since CD73 expression is high in basal B breast cancer cells (Figure 2A), we included MDA-MB-231 and Hs578T basal B cells as positive Ctrls. Baseline signals were similar for MCF-7 Ctrl, GRHL2 KO-1 and KO-2, and the two positive Ctrls, but the addition of AMP as a substrate significantly increased adenosine production in the positive Ctrl cells and the GRHL2-depleted MCF-7 cells, while no significant increase was found for the MCF-7 Ctrl cells (Figure 4C). To demonstrate that the increase was due to the activity of CD73, we made use of an enzymatic inhibitor of CD73, α,β-methylene ADP (APCP). Indeed, APCP prevented the conversion of AMP into adenosine in the positive Ctrl cells and the GRHL2-depleted MCF-7 cells. These data showed that, in agreement with the negative regulation of NT5E/CD73 by GRHL2, loss of GRHL2 triggers increased CD73-mediated adenosine production.

### Enhanced CD8^+^ T cell recruitment due to CD73-mediated adenosine production in response to GRHL2 loss

**Figure 5:**
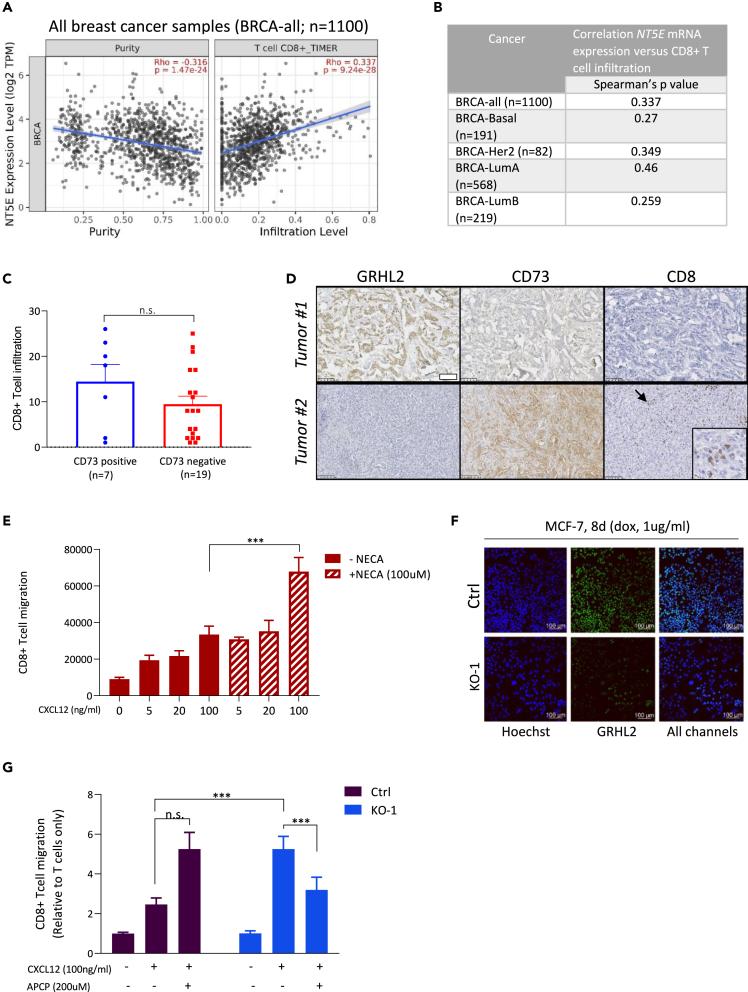
Enhanced CD8^+^ T cell recruitment in response to GRHL2 loss (A) TIMER 2.0 scatterplots showing the correlation of NT5E mRNA expression with tumor purity (percentage of malignant cells in a tumor tissue; left) and the predicted presence of CD8^+^ T cells (right) in all breast cancer (BRCA) lesions tested. (B) Table showing TIMER 2.0 Spearman correlation scores for association between NT5E mRNA and predicted presence of CD8^+^ T cells across breast cancer patient tumors separated for different molecular subtypes. (C) CD8 score (% CD8 positive T cells) derived from IHC, on whole slides of a series of metaplastic (TNBC) and high and low GRHL2 mRNA tumors, separated for IHC determined CD73 membrane staining positive and negative cases. Mean and SEM is shown. *p* value calculated using non-parametric t test with unequal variance. (D) Representative IHC images for GRHL2, CD73, and CD8 in breast cancer tissues. Arrow indicates area of infiltrated CD8 T cells in tumor 2 that is enlarged in the lower right corner. Bar, 50 μm. (E) Quantification of the number of CD8^+^ T cells recruited toward the lower compartment of transwells at increasing concentrations of CXCL12 in presence or absence of 100 μM stable adenosine analog, NECA. Average and SEM of 3 biological replicates is shown (two-way ANOVA test; ∗∗∗*p* < 0.001. (F) Immunofluorescence staining showing GRHL2 loss at 8 days after doxycycline-mediated Cas9 induction in sgGRHL2 cells but not sgCtrl cells for the experiment shown in G. Green, GRHL2 Ab; Blue, Hoechst. (G) Quantification of CD8^+^ T cells recruited toward the lower compartment of transwells seeded with control or GRHL2 KO-1 MCF-7 cells (8 days 1 μg/ml doxycycline treatment) in absence or presence of 100 ng/mL CXCL12 and 200 μM APCP CD73 inhibitor. Data normalized to the T cells only condition. Mean and SEM of 3 biological replicates is shown (two-way ANOVA test; ∗∗∗*p* < 0.001; n.s., non-significant).

Adenosine is known to suppress the activation, proliferation, and effector differentiation of CD8 T cells.^9^^,^^10^^,^^11^^,^^44^ Yet, for melanoma and bladder cancer it has been observed that T cell-specific loss of A2AR adenosine receptors leads to a reduction in tumor-associated CD8^+^ T cells, indicating that adenosine does not interfere with T cell recruitment in solid tumors.^45^ To address the relation between CD73 expression and CD8 T cell recruitment in breast cancer, we examined the association NT5E mRNA levels and CD8 T cell infiltration using the TIMER 2.0 patient dataset.^46^ Expression of NT5E mRNA was positively associated with CD8 T cell infiltration in all breast cancer subtypes tested (Figure 5A and 5B). We next evaluated the correlation between GRHL2, CD73, and CD8 by IHC in a set of 10 GRHL2-high, 10 GRHL2-low (based on RNA-seq), and 10 metaplastic breast adenocarcinoma patient tumors. Again, there was a trend toward lower CD8 infiltration in CD73-negative tumors although CD8 IHC was heterogeneous and in this small set of tumors the difference with CD73 positive tumors was not significant (Figures 5C and 5D; Figure S2). This raised the possibility that enhanced CD73-mediated adenosine production by tumor cells may in fact stimulate rather than inhibit T cell recruitment (while locally suppressing T cell function as demonstrated by others). We therefore examined how GRHL2 deletion, by increasing CD73 expression and adenosine production, affected recruitment of CD8+ T cells to tumor cells using the MCF-7 conditional KO model.

We first measured CD8^+^ T cell migration in the presence of a known chemoattractant, CXCL12, in the absence or presence of a stable adenosine analog (NECA) using a transwell assay. CXCL12 stimulated CD8^+^ T cell migration in a concentration-dependent fashion, which was further increased by NECA (Figure 5E; Figure S3). At 100 ng/ml CXCL12 the presence of NECA significantly increased CD8^+^ T cell migration (*p* < 0.0001). Next, a similar setup was used to investigate the impact of seeding MCF-7 cells expressing Ctrl sgRNA or GRHL2 sgRNA cells at the bottom of the transwell system and inducing GRHL2 depletion by incubating with doxycycline for 8 days (Figures 5F and 5G; Figure S4). No significant impact of Ctrl or GRHL2 KO cells was observed on CD8^+^ T cell migration in the absence of CXCL12. In the presence of CXCL12, the co-culture with GRHL2 KO cells stimulated CD8^+^ T cell migration more strongly as compared to the co-culture with Ctrl cells. To assess if the increase in CD8+T cell migration in the presence of GRHL2 KO cells was due to CD73-mediated adenosine production, we made use of the APCP CD73 inhibitor. Notably, while APCP did not inhibit the CD8 T cell migration in the presence of Ctrl MCF-7 cells, it attenuated the enhanced migration of CD8^+^ T cells toward GRHL2 KO cells (Figure 5G; Figure S4).

Altogether, these results indicated that CD73-mediated adenosine production is associated with increased CD8^+^ T cell recruitment in breast tumors. Loss of GRHL2, through enhanced CD73 expression, stimulates CD8^+^ T cell migration toward breast cancer cells.

## Discussion

*GRHL2* is located on chromosome 8q22 that is frequently amplified in carcinomas.^47^ Yet, tumors are heterogeneous and our current work and that of others^39^ show that GRHL2 expression is variable in breast cancer tissues. Our findings indicate that loss of GRHL2 can contribute to enhanced extracellular adenosine production in the TME, which has been implicated in suppression of the activation, proliferation, and effector differentiation of NK cells and T cells.^9^^,^^11^

In the patient data as well as in the cell line panel the correlation between GRHL2 and NT5E/CD73 is heterogeneous. We observe a significant inverse correlation, but there are examples where both GRHL2 and NT5E/CD73 are present, or both are absent. This indicates that other mechanisms regulate the expression of NT5E/CD73 besides GRHL2. Indeed, several additional mechanisms impinging on CD73 have been reported. EMT can modulate CD73, which may involve activation of TGF-β signaling and the SNAIL transcription factor, and this may contribute to immune suppression.^30^^,^^31^^,^^32^ We have previously shown that, in the MCF-7 conditional KO model used in this study, deletion of GRHL2 has limited impact on EMT markers,^40^^,^^41^ suggesting that enhanced CD73 expression and adenosine production are not a consequence of EMT in this case. Increased transcription of NT5E through activation of hypoxia-inducible factor-1 (HIF-1) or loss of hormone receptors such as estrogen receptor (ER) has also been identified as alternative mechanisms regulating CD73 expression.^48^^,^^49^^,^^50^ However, we have not observed activation of the HIF pathway or changes in ER⍺ expression upon loss of GRHL2.^41^ GRHL2 has been reported to act as a pioneer factor, promoting chromatin accessibility, and GRHL2 has been found to co-occupy enhancer elements with FOXA1, GATA3, and ER⍺ in hormone receptor-positive breast cancers. However, we have previously found that only a minor proportion of GRHL2 binding sites in the genome of breast cancer cell lines coincide with ER⍺ binding sites,^41^ and in our current study no ER⍺ motif was identified in the vicinity of the GRHL2 peak in the NT5E gene. Our findings point to an inhibitory interaction of GRHL2 with the NT5E gene at a conserved binding site located in an intronic region. Here, GRHL2 may interact with enhancer elements or regulate histone modifications,^51^^,^^52^ similar to what we and others have observed for the majority of GRHL2 target genes.^41^^,^^51^^,^^53^ Our finding that NT5E/CD73 is inversely correlated with GRHL2 in breast cancer cell lines and in patient tumors or tumor areas suggests the negative regulation of CD73 by GRHL2 in breast cancer is common. Notably, in the scoring of CD73 in patient tissues we have focused on membrane-associated CD73. The impact of alterations in CD73 expression may be further determined by the localization of CD73, a GPI-linked protein that can be membrane bound or shed from the membrane.^13^

GRHL2 was previously shown to support NK cell-mediated tumor cytotoxicity through a mechanism involving epigenetic stimulation of ICAM-1 expression, which supported enhanced NK-target cell synapse formation.^54^ However, GRHL2 has not been previously implicated in the interaction of tumor cells with T cells. The elevated CD73-mediated extracellular adenosine levels triggered by the loss of GRHL2 are expected to suppress T cell activity based on earlier reports. Adenosine binds to GPCRs on immune cells thereby stimulating (via A2A and A2B receptors) or suppressing (via A1 and A3 receptors) adenylyl cyclase activity, stimulating intracellular calcium release (via A1 and A3 receptors), and activating ERK and p38 mitogen-activated protein kinase (MAPK) signaling (via A1, A2A, A2B, A3 receptors).^9^ Stimulation of A2AR on T cells has been reported to suppress the activation, proliferation, and effector differentiation of CD8 T cells,^44^ and A2AR deletion in mouse models leads to enhanced tumor killing by CD8 cells.^55^ Indeed, CD73 represents an actionable target to reduce extracellular adenosine levels and enhance anti-tumor immunity.^16^ Our current work, however, shows that enhanced CD73-mediated extracellular adenosine production may stimulate rather than inhibit recruitment of CD8 T cells, and we corroborate this effect using a stable adenosine analog. Interestingly, there is precedent for a pro-migratory effect of adenosine in different contexts. Adenosine has been implicated in the migration of dendritic cells toward regulatory T cells,^56^ and T cell-specific deletion or pharmacological inhibition of A2AR has been shown to cause increased growth of ectopic tumors in mice as expected but in fact led to reduced CD8 T cell accumulation in these tumors.^45^ Our finding that CD73 expression is not negatively correlated with CD8 T cell infiltration but rather associates with a slight increase further establishes a dual role of extracellular adenosine in T cell migration versus cytotoxic activity.

In summary, this work demonstrates that GRHL2, a key epithelial transcriptional regulator, can regulate extracellular adenosine levels produced by breast cancer cells through suppression of the gene encoding the NT5E/CD73 ecto-enzyme. Moreover, this study shows that adenosine produced by GRHL2-depleted breast cancer cells (or provided as a stable analog) can augment the recruitment of CD8 T cells, in addition to its previously established role as a suppressor of T cell-mediated cytotoxicity.

### Limitations of the study

We focused on the impact of the GRHL2-NT5E/CD73-adenosine axis on T cell migration. The suppression of T cell-mediated cytotoxicity by adenosine has been firmly established by others.^9^^,^^10^^,^^11^^,^^16^^,^^44^ The MCF-7 conditional KO model shows decreased proliferation from 2 days after GRHL2 deletion followed by a minor decrease in viability after 1 week (in agreement with the established roles of GRHL2).^35^^,^^40^ This precludes use of the model in assessment of T cell-mediated cytotoxicity (e.g., in the context of biAbs).

CD73-mediated production of adenosine can involve tumor cells, stromal cells, regulatory T cells, and extracellular vesicles derived from these cell types.^57^^,^^58^^,^^59^ Our study demonstrates that GRHL2 levels can contribute to modulation of adenosine production by tumor cells, but further (*in vivo*) work is required to reveal the relative contribution of adenosine produced by GRHL2-negative tumor cells in such a complex environment.

## STAR★Methods

### Key resources table

**Table:** 

REAGENT or RESOURCE	SOURCE	IDENTIFIER
**Antibodies**		

Anti-GRHL2 antibody Clone CL3760	Atlas/Sigma	Cat# AMAb91226
Anti-5′-Nucleotidase Clone 4G6E3	Novus Bio	Cat# NBP2-37271
Liquid DAB+, 2-component system	Dako Agilent	Cat# K346811-2
EnVision+/HRP, Mouse	Dako Agilent	Cat# K400111-2
Rabbit-anti-human GRHL2 antibody	Atlas/Sigma	Cat# hpa004820
AlexaFluor-488 conjugated anti-rabbit secondary antibody	Mol Probes	Cat# A11008
HRP-conjugated anti-mouse secondary antibody	Jackson Immunoresearch	Cat# 115-035-003
HRP-conjugated anti-rabbit secondary antibody	Jackson Immunoresearch	Cat# 111-035-003
Alexa 647-linked anti-mouse secondary antibody	Jackson Immunoresearch	Cat# 115-605-146
Anti-α-Tubulin antibody	Sigma	Cat# T-9026
Fixable Viability Dye efluor 780	Invitrogen	Cat# 65-0865-14
Anti-CD8 antibody	Biolegend	Cat# 344731
Anti-GAPDH antibody	Santa Cruz	Cat# sc-32233
Anti-CD73 antibody [4G6E3]	Abcam	Cat# ab202122
Anti-Cas9 (S. pyogenes) antibody (7A9-3A3)	Cell Signaling	Cat# 14697S

**Biological samples**		

Breast cancer primary tumors	Erasmus University Medical Center	MEC 02-953

**Chemicals, peptides, and recombinant proteins**		

Fetal Bovine Serum	PAN-Biotech	Cat# P30-3301
Blasticidin	Gibco	Cat# R21001
Puromycin	Santa Cruz	Cat# sc-108071A
Doxycycline	Selleckchem	Cat# 8634-1/S5159
Target Retrieval Solution, Citrate pH6	Dako Agilent	Cat# S236984-2
Protein Block, Serum-free solution	Dako Agilent	Cat# X090930-02
Antibody Diluent	Dako Agilent	Cat# S302283-2
Bovine Serum Albumin	Merck/Sigma	Cat# A9647
Hoechst 33258	Sigma	Cat# 861405
TRI reagent	Invitrogen	Cat# AM9738
Adenosine 5'-(α,β-methylene) diphosphate sodium salt	Santa Cruz	Cat# sc-214457
Adenosine 5′-monophosphate sodium salt	Sigma	Cat# A1752
Histopaque®-1077	Sigma	Cat# 10771
Trypan blue	Biorad	Cat# 1450021
ECL (Prime) Western Blotting Detection Reagent	GE Healthcare	Cat# 12316992
Cell Tracker™ Green CMFDA Dye	Invitrogen	Cat# C2925

**Critical commercial assays**		

RevertAid H minus first-strand cDNA synthesis kit	Thermo Scientific	Cat# K1632
Malachite green phosphate detection kit	R&D Systems	Cat# DY966
ECL (Prime) Western Blotting Detection Reagent	GE Healthcare	Cat# 12316992
CD8^+^ T cell Isolation Kit	Miltenyi Biotec	Cat# 130-096-495

**Deposited data**		

GRHL2 Bru-seq data	GEO Database	Accession No. GSE222353
GRHL2 ChIP-seq data	USCS Genome Browser	https://genome.ucsc.edu/s/hwuRadboudumc/ZWang
GRHL2 motif data	Jaspar database	https://jaspar.genereg.net/
MEME ChIP and FIMO analysis	MEME Suite 5.5.3	https://meme-suite.org/meme/
RNA seq. of Breast adenocarcinoma cell lines	Zenodo	https://zenodo.org/record/4560297/export/xm#.YVXHC5pBxQA
METABRIC dataset	cBioPortal	https://www.cbioportal.org/
CD8+T cell infiltration data	Timer 2.0	http://timer.cistrome.org/

**Experimental models: Cell lines**		

MCF-7	ATCC	ATCC HTB-22
CAMA-1	ATCC	ATCC HTB-21
T47D	ATCC	ATCC HTB-133
MDA-MB-361	ATCC	ATCC HTB-27
BT474	ATCC	ATCC HTB-20
HCC1806	ATCC	ATCC CRL-2335
HCC1143	ATCC	ATCC CRL-2321
MDA-MB-231	ATCC	ATCC CRM-HTB-126
BT-549	ATCC	ATCC HTB-122
Hs578T	ATCC	ATCC HTB-126

**Oligonucleotides**		

sgGRHL2/1 (KO-1): CCTCGAGACAAGAGGCTGCTGTC	Sigma	Sanger Arrayed Whole Genome Lentiviral CRISPR Library
SgGRHL2/2 (KO-2): AAATTTCGGAGTGCTTCAGTTGG	Sigma	Sanger Arrayed Whole Genome Lentiviral CRISPR Library
Primer: GRHL2 F: GGCAGTGTCCTTGTTAAACGG Primer: GRHL2 R: ATCGTCAGTCTCCTTCCTCACG	This paper	N/A
Primer: NT5E F: CTCCTCTCAATCATGCCGCT Primer: NT5E R: CCCAGGTAATTGTGCCATTGT	This paper	N/A
Primer: Actin F: ATTGCCGACAGGATGCAGAA Primer: Actin R: GCTGATCCACATCTGCTGGAA	This paper	N/A

**Recombinant DNA**		

Lentiviral Edit-R inducible cas9 plasmid	Dharmacon	Cat# VCAS11227

**Software and algorithms**		

FlowJo 10.8.1	BD Biosciences	
GraphPad Prism v8	GraphPad software Inc.	https://www.graphpad.com/features

### Resource availability

#### Lead contact

Erik HJ Danen (e.danen@lacdr.leidenuniv.nl)

#### Materials availability

The figures and supplemental information contain the data. No additional materials were generated as part of this study.

#### Data and code availability

Data: All data generated in the study is presented in this published article.

Code: This paper does not report original code.

The lead contact can provide the information for all relevant data and resources upon request.

### Experimental model and study participant details

#### Human subjects

The Erasmus MC medical ethics committee has declared that retrospective biomarker research on surgically resected specimen is according to Dutch law exempt of medical ethical approval (MEC 02–953). Furthermore, the study has been performed in accordance with the national guidelines of the FeDeRa, the Federation of Bio-medical research communities. (https://www.coreon.org/wp-content/uploads/2020/04/coreon-codegoedgebruik-versie-4juli2015.pdf). All surgically resected specimen were obtained from female breast cancer patients. Blood samples were obtained from the Centre for Human Drug Research, Leiden, NL. Blood was collected from healthy male and female donors after obtaining written informed consent in accordance with Good Clinical Practice guidelines and the Declaration of Helsinki.

### Method details

#### Cell culture

The human breast adenocarcinoma cell lines MCF-7, CAMA-1, T47D, MDA-MB-361, BT474, HCC1806, HCC1143, MDA-MB-231, BT-549, and Hs578T were grown in RPMI 1640 (Gibco, Fisher Scientific, Landsmeer, The Netherlands) supplemented with 10% fetal bovine serum (FBS), 25 U/mL penicillin, and 25 μg/mL streptomycin (PAN Biotech) in a humidified incubator with 5% CO2 at 37 °C.

#### Conditional KO procedure

Inducible Cas9 expression was generated by lentiviral transduction with Lentiviral Edit-R inducible Cas9 plasmid (Dharmacon) in MCF-7 cells. Single-cell selection was performed using 2 μg/mL blasticidin. Inducible MCF-7-Cas9 cells were then transduced with a non-targeting sgRNA and two sgRNAs targeting GRHL2 to generate inducible GRHL2 knockout cells. (Sanger Arrayed Whole Genome Lentiviral CRISPR Library (Sigma–Aldrich); sgGRHL2/1 (KO-1): CCTCGAGACAAGAGGCTGCTGTC, sgGRHL2/2 (KO-2): AAATTTCGGAGTGCTTCAGTTGG). Bulk selection was performed using 8 μg/mL puromycin for 72hrs and induction of GRHL2 KO by doxycycline treatment was verified.

#### ChIP-seq, motif analysis, and Bru-seq

The visualization of GRHL2 binding along the NT5E gene was obtained using the UCSC Genome Browser. ChiP-seq data supporting the results of this article is available at the UCSC Genome Browser [https://genome.ucsc.edu/s/hwuRadboudumc/ZWang]. Two different binding profiles of GRHL2 were retrieved from the JASPAR database (https://jaspar.genereg.net/). A motif discovery analysis was performed on the NT5E GRHL2 ChIP-seq peaks using the MEME-ChIP tool^60^ from the MEME Suite 5.5.3 with default settings. The FIMO tool^43^ was used to scan the NT5E gene for occurrences of the AACC[A/C/G]GTT conserved GRHL2 motif.^60^

Bru-seq data supporting the results of this article is available at Gene Expression Omnibus (GEO) database, www.ncbi.nlm.nih.gov/geo (Accession No. GSE222353).

#### RNA analysis in cell lines and clinical samples

GRHL2 and NT5E gene expression was analyzed using RNA sequencing data from a panel of 52 human breast adenocarcinoma cell lines representing luminal, basal A, and basal B subtypes (https://zenodo.org/record/4560297/export/xm#.YVXHC5pBxQA). Log2 normalized gene expression values were used for further analyzing. Violin plots were made by GraphPad.

Co-expression analysis in clinical samples was performed using cBioPortal (https://www.cbioportal.org/) database. The mRNA expression data of breast cancer patients were retrieved from the METABRIC dataset,^61^^,^^62^ consisting of 1904 targeted sequenced tumor samples and visualized in cBioPortal. Co-expression z-scores (Pearson and Spearman values) were calculated on the cBioPortal website.

#### IHC on samples of human breast cancer tissue and cell lines

Formalin-fixed paraffin-embedded (FFPE) primary metaplastic tumors (*n* = 10) and primary tumors with high (*n* = 10) or low (*n* = 10) GRHL2 RNA levels were collected from breast cancer patients who entered the Erasmus University Medical Center (Rotterdam, The Netherlands) for local treatment of their primary disease between 1985 and 2005. Sections of 4 μm were cut, pasted on starfrost adhesive slides, and dried overnight at 37°C. Slides were dewaxed with Xylene solution and hydrated with decreasing alcohol percentages (100%, 95%, 75% and 50%). Antigen retrieval was done using Target Retrieval Solution, Citrate pH6 (Dako Agilent, S236984-2) or Target Retrieval buffer, pH6 (S1699; for GRHL2) or pH9 (2367; for CD73 and CD8) for 20 min at 95°C followed by 20 min at room temperature. Subsequently, slides were immersed in 3% hydrogen peroxide/PBS for 10 min to block endogenous peroxidase activity. To block non-specific binding sites, a Protein Block, Serum-free solution (Dako Agilent, X090930-02) was used for 30 min. Then, slides were incubated for 1 h with, 1:1250 mouse-*anti*-human GRHL2 antibody (Clone CL3760; Cat.nr AMAb91226; Atlas/Sigma), 1:400 mouse-*anti*-human 5′-Nucleotidase/CD73 (Clone 4G6E3; Cat.nr. NBP2-37271, Novusbio), or 1:200 mouse-*anti*-human CD8 antibodies (Clone C8/144B; Cat.nr M7103; Dako Agilent), all diluted with Antibody Diluent (S302283-2, Dako Agilent). Negative controls were made by replacing the primary antibody with mouse immunoglobulin at identical dilution. Detection and visualization of the antibodies was done with EnVision+ Single reagent HRP, Mouse (Dako Agilent, K4001) and Liquid DAB+, 2-component system (Dako Agilent, K346811-2). Slides were counterstained with hematoxylin and dehydrated through graded alcohol and xylene and cover slides were mounted with Pertex. Results of positive control tissues of liver, skin, and intestine were compared with results in human protein atlas for the antibodies used. Results were comparable. Images were made using Nikon ECLIPSE E4000. GRHL2 was scored for absence or presence of nuclear staining in breast tumor cells. CD73 was scored for negative or weak staining (0), weak or partial membrane staining (0.5), or clear positive membrane staining (1) in breast tumor cells. CD8 was scored for the % of CD8 positive cells in the tumor area.

#### Immunofluorescence

Cells were fixed and permeabilized by incubation with 4% formaldehyde and 0.1% Triton X-100 in Phosphate-buffered saline (PBS) for 15 min, followed by a blocking step with 0.5% w/v bovine serum albumin (BSA, Sigma Aldrich) in (PBS) for 30 min. Then, cells were incubated with the GRHL2 primary antibody (1:500, Atlas-Antibodies, hpa004820) in 0.5% w/v BSA in PBS overnight at 4°C. The cells were washed three times in PBS supplemented with 0.5% BSA (BSA-PBS) and subsequently stained with Alexa Fluor 488 conjugated anti-rabbit secondary antibody (1:1000, Mol Probes, A11008) and Hoechst 33258 (1:10,000, Sigma Aldrich, 861405) for 1 h at room temperature, followed by three times washing steps with 0.5% BSA-PBS. Cell preparations were imaged using a Nikon ECLIPSE Ti2 confocal microscope.

#### Western blotting and flow cytometry

Cells were lysed for total protein isolation and protein samples (20 μg/lane) were run on sodium dodecyl sulfate–polyacrylamide gel electrophoresis (SDS-PAGE). The gels were transferred onto a polyvinylidene difluoride (PVDF) membrane (Millipore) followed by blocking with 5% BSA in Tris-buffered saline (TBS), with 0.05% Tween 20 (TBS-T). Membranes were incubated with GRHL2 (Atlas-Antibodies, hpa004820), cas9 (Cell Signaling, 14697S), CD73 (Abcam, ab202122), and α-tubulin (Sigma Aldrich, T-9026) antibodies at 4°C, overnight. Horseradish peroxidase-conjugated secondary antibodies (Jackson Immunoresearch, anti-rabbit 111-035-003, anti-mouse 115-035-003) and Alexa 647-linked anti-mouse (Jackson Immunoresearch, 115-605-146) were used at room temperature, 1h. Primary and secondary antibodies were prepared in 1% BSA in TBS-T. Signals were detected with an Amersham Imager 600 (GE Healthcare Life Sciences, Eindhoven, The Netherlands) using Cy5 fluorescence and ECL (Prime) Western Blotting Detection Reagent (GE Healthcare Life Sciences). Band intensities were quantified with ImageJ.

For flow cytometry, cells were detached with 0.02% EDTA. Surface expression levels were determined using CD73 (Clone 4G6E3; Cat.nr. NBP2-37271, Novusbio), CD8 (Clone SK1; Cat.nr 344702; Biolegend), or isotype control primary antibodies, followed by fluorescence-conjugated secondary antibodies. For analysis of T cells the CD8 staining was combined with a live/dead stain (fixable viability dye, Invitrogen). Samples were measured on a CytoFLEX S (Beckman Coulter) and analyzed using FlowJo (version 10).

#### qRT-PCR

Total RNA was extracted using Trizol (TRI reagent, AM9738, Invitrogen). cDNA was synthesized using the RevertAid H minus first-strand cDNA synthesis kit (Thermo Fisher Scientific) according to the manufacturer’s protocol. Gene expression was normalized to the Actin gene and further analyzed with the 2−ΔΔCt method.

#### Malachite Green Assay

GRHL2 KO was induced in MCF-7 Ctrl, KO-1, KO-2 cells with 1 μg/mL doxycycline for 7 days. On day 7, MCF-7 cells together with Hs578T and MDA-MB-231 were seeded as 25.000 cells per well in triplicate in a 96-well plate. Cell media was discarded 24h later and the cells were washed with phosphate-free buffer (2 mM MgCl2,1 mM KCl,10 mM glucose, 125 mM NaCl, 10 mM HEPES pH 7.2, diluted in ddH2O). Then, cells were treated with a CD73 inhibitor Adenosine 5'-(α,β-methylene) diphosphate (APCP) (25μM final concentration; Sigma Aldrich) for 15 min at 37°C. Adenosine monophosphate (AMP; 100 μM final; Sigma Aldrich) was added and incubated for 110 min at 37°C. Only phosphate-free buffer was added to the wells indicated as the control condition. Inorganic phosphate production in the cell supernatants was measured using the malachite green phosphate detection kit (R&D Systems) following the manufacturer’s instructions.

#### *Trans*-well T cell migration assay and flow cytometry

MCF-7 Ctrl, KO-1, and KO-2 cells were seeded in 24-well plates (20,000 cells per well) after 5 days of doxycycline treatment (1 μg/mL). Human peripheral blood mononuclear cells (PBMCs) were isolated from peripheral blood from healthy volunteers by Histopaque-1077 (10771, Sigma-Aldrich) density gradient centrifugation and enriched for CD8^+^ T cells with the CD8^+^ T cell Isolation Kit (130-096-495, Miltenyi Biotec) according to manufacturer’s instructions. Enriched CD8^+^ T cells were stained with Cell Tracker Green CMFDA Dye (1uM) (C2925, Invitrogen) for 45 min and added (200,000 cells per transwell) on top of the filter membrane of the transwell insert (6.5 mm Transwell with 3.0 μm pore, Corning). The cancer cells and CD8+T cells were cultured with RPMI 1640, supplemented with 10% human plasma isolated freshly from the healthy blood donors, 25 U/ml penicillin, 25 μg/mL Streptomycin at 37°C in a humidified 5% CO_2_ incubator. After 48 h, filters were discarded and 20 fluorescence (488nm) and phase-contrast images per well were taken with 20x magnitude imaging using ZOE Fluorescent Cell Imager. The supernatant from the lower chamber was also collected to quantify migrated T cells by using a hemocytometer after Trypan blue staining (1450021, Biorad).

### Quantification and statistical analyses

Data were represented as the mean ± standard deviation (SD) or means ± standard error of the mean (SEM) of at least two independent experiments. Two-way analysis of variance (ANOVA) with Bonferroni’s multiple comparison test was performed unless otherwise stated. Non-parametric t-test with unequal variance was employed to determine statistical significance of the difference between groups in IHC experiments. Statistical data were obtained using GraphPad Prism 8 (GraphPad Software, La Jolla, CA, USA). ns, not significant; ∗*p* < 0.05, ∗∗*p* < 0.01, ∗∗∗*p* < 0.001 and ∗∗∗∗*p* < 0.0001.
