# Supplementary material for: GRHL2 suppression of NT5E/CD73 in breast cancer cells modulates CD73-mediated adenosine production and T cell recruitment
Source: iScience. 2024 Apr 12;27(5):109738. doi: 10.1016/j.isci.2024.109738 (PMC11068632; doi:10.1016/j.isci.2024.109738)
Supplement: Document S1. Figures S1–S4 [file mmc1.pdf]

iScience, Volume *27*

Supplemental information

GRHL2 suppression of NT5E/CD73 in breast cancer cells modulates CD73-mediated adenosine production and T cell recruitment

Bircan Coban, Zi Wang, Chen-yi Liao, Klara Beslmüller, Mieke A.M. Timmermans, John W.M. Martens, Jasmijn H.M. Hundscheid, Bram Slutter, Annelien J.M. Zweemer, Elsa Neubert, and Erik H.J. Danen

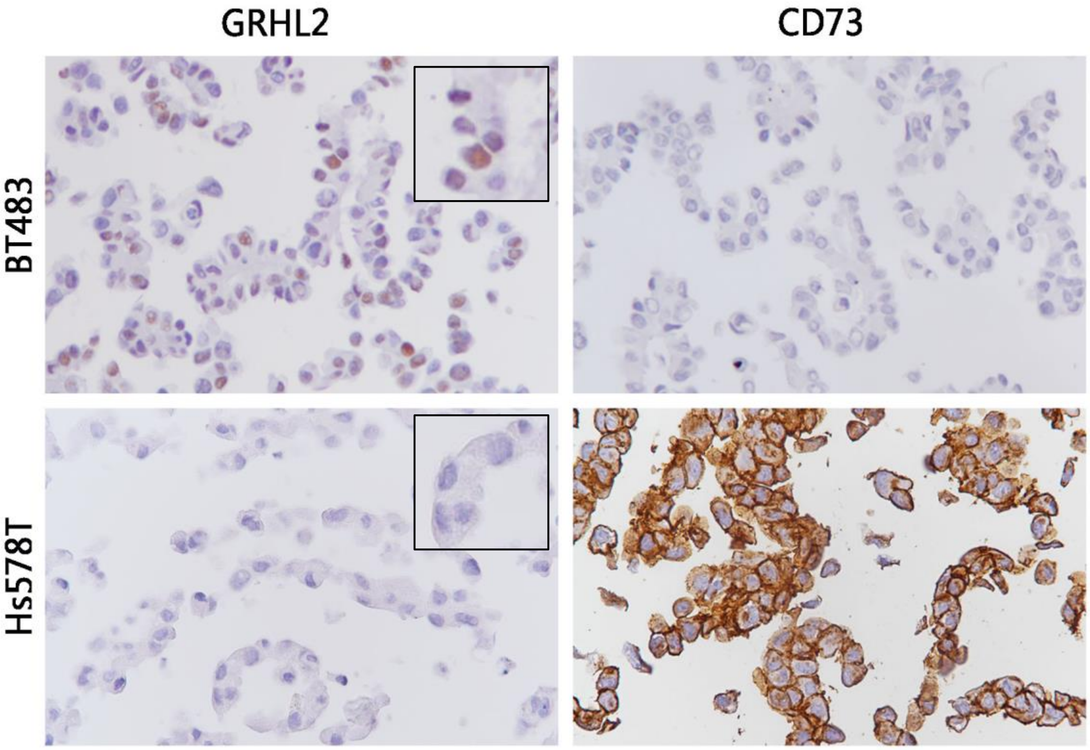

Coban et al., Figure S1

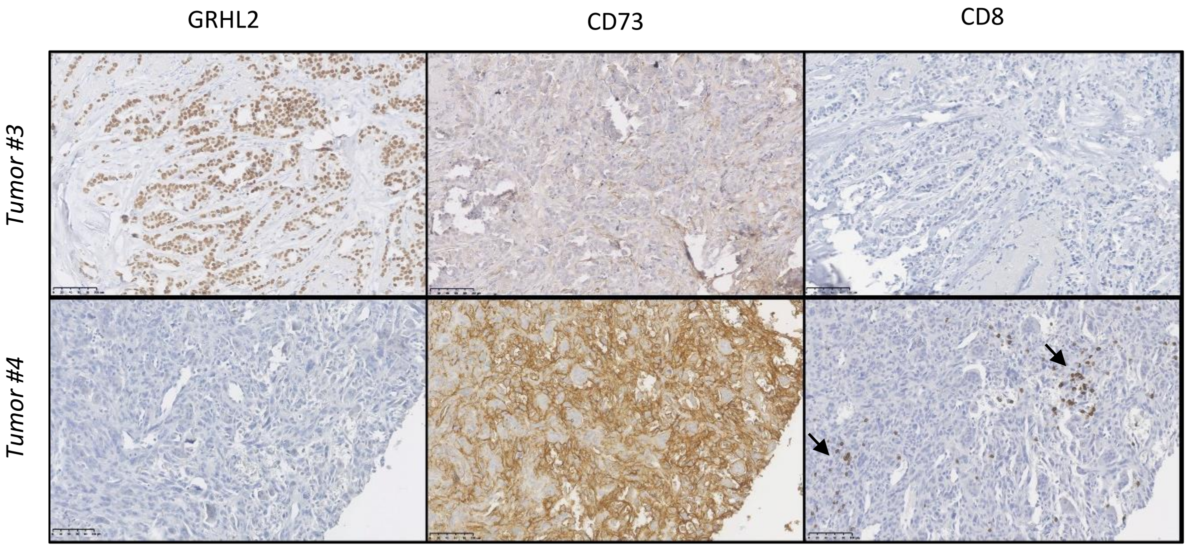

Coban et al., Figure S2

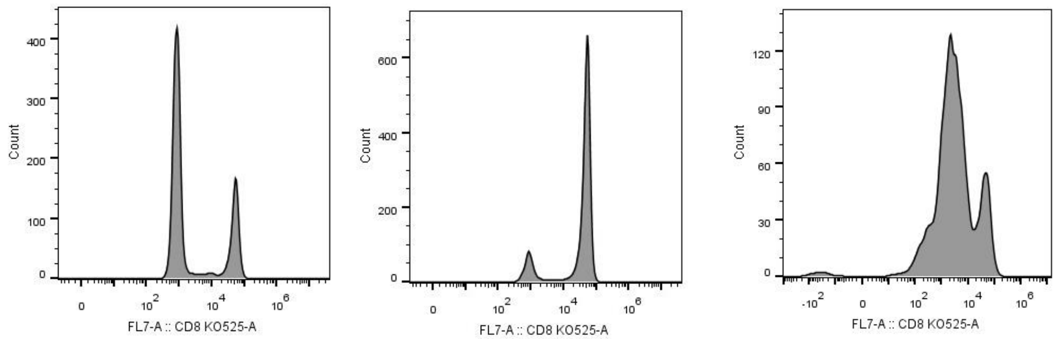

PBMC

MACS sorted

İnput

Migrated cells

Output

Coban et al., Figure S3

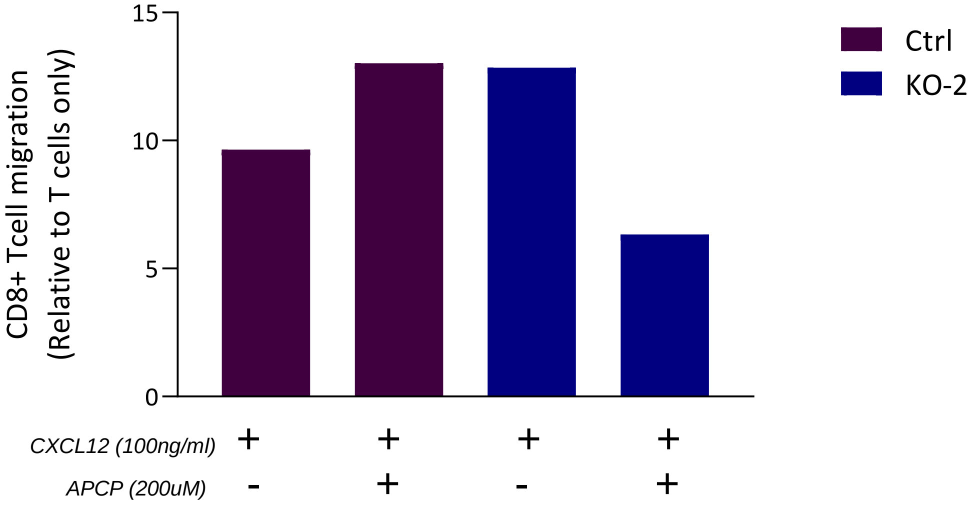

Coban et al., Figure S4

Figure S1: GRHL2 and CD73 protein expression in breast cancer cell lines (related to Figure 2).

GRHL2 and CD73 IHC results for a luminal cell line, showing nuclear GRHL2 expression (BT483) versus a basal B cell line lacking GRHL2 (Hs578T).

Figure S2: IHC for GRHL2, CD73, and CD8 in breast cancer lesions (related to Figure 5C,D). Representative IHC images for GRHL2, CD73, and CD8 in breast cancer tissues. Arrows indicate infiltrated CD8 T cells in tumor 4.

Figure S3: Flow cytometry analysis of CD8 T cells in trans-well assay (related to Figure 5E).

CD8 surface expression determined by flow cytometry for PBMCs (left), purified CD8 T cells that were added to the upper compartment of trans-wells (middle), and for the T cells migrated to the bottom compartment in presence of CXCL12 (right).

Figure S4: CD8 T cell recruitment in presence of control or GRHL2 KO MCF-7 cells (related to Figure 5G).

Quantification of CD8+ T cells recruited towards the lower compartment of trans-wells seeded with control or GRHL2 KO-2 MCF-7 cells (8 days 1ug/ml doxycycline treatment) in absence or presence of 100 ng/ml CXCL12 and 200uM APCP CD73 inhibitor. Data normalized to the T cells only condition. One experiment is shown.
